# New Perspectives in the Renin-Angiotensin-Aldosterone System (RAAS) III: Endogenous Inhibition of Angiotensin Converting Enzyme (ACE) Provides Protection against Cardiovascular Diseases

**DOI:** 10.1371/journal.pone.0093719

**Published:** 2014-04-01

**Authors:** Miklós Fagyas, Katalin Úri, Ivetta M. Siket, Andrea Daragó, Judit Boczán, Emese Bányai, István Édes, Zoltán Papp, Attila Tóth

**Affiliations:** 1 Division of Clinical Physiology, Institute of Cardiology, University of Debrecen, Debrecen, Hungary; 2 Department of Neurology, University of Debrecen, Debrecen, Hungary; 3 Institute of Internal Medicine, Division of Nephrology, University of Debrecen, Debrecen, Hungary; School of Pharmacy, Texas Tech University HSC, United States of America

## Abstract

ACE inhibitor drugs decrease mortality by up to one-fifth in cardiovascular patients. Surprisingly, there are reports dating back to 1979 suggesting the existence of endogenous ACE inhibitors. Here we investigated the clinical significance of this potential endogenous ACE inhibition.

ACE concentration and activity was measured in patient's serum samples (n = 151). ACE concentration was found to be in a wide range (47–288 ng/mL). ACE activity decreased with the increasing concentration of the serum albumin (HSA): ACE activity was 56±1 U/L in the presence of 2.4±0.3 mg/mL HSA, compared to 39±1 U/L in the presence of 12±1 mg/mL HSA (values are mean±SEM). Effects of the differences in ACE concentration were suppressed in human sera: patients with ACE DD genotype exhibited a 64% higher serum ACE concentration (range, 74–288 ng/mL, median, 155.2 ng/mL, n = 52) compared to patients with II genotype (range, 47–194 ng/mL, median, 94.5 ng/mL, n = 28) while the difference in ACE activities was only 32% (range, 27.3–59.8 U/L, median, 43.11 U/L, and range 15.6–55.4 U/L, median, 32.74 U/L, respectively) in the presence of 12±1 mg/mL HSA. No correlations were found between serum ACE concentration (or genotype) and cardiovascular diseases, in accordance with the proposed suppressed physiological ACE activities by HSA (concentration in the sera of these patients: 48.5±0.5 mg/mL) or other endogenous inhibitors.

Main implications are that (1) physiological ACE activity can be stabilized at a low level by endogenous ACE inhibitors, such as HSA; (2) angiotensin II elimination may have a significant role in angiotensin II related pathologies.

## Introduction

The renin-angiotensin-aldosterone system (RAAS) is an important regulator of blood pressure and salt-water homeostasis. Angiotensin converting enzyme is a member of RAAS [1], which catalyses the cleavage of angiotensin I to angiotensin II, and takes part in the metabolism of other peptides like bradykinin [2].

ACE is a prime target in the treatment of common diseases, hereby ACE inhibitors represent one of the most commonly used drugs. This is supported by the fact, that there were 162.8 million ACE inhibitor prescriptions in 2009 in the United States of America [3], which will probably increase during the next years. Their effectiveness is proven by several large clinical trials: ACE inhibitors reduce the risk of cardiovascular death, nonfatal myocardial infarction or cardiac arrest in stable coronary heart disease [4], improve the prognosis [5] and reduce the 5-week mortality after myocardial infarction [6], reduce heart failure mortality [7], inhibit left ventricular remodeling [8], delay the manifestation of hypertension [9], and reduce the left ventricular mass index in left ventricular hypertrophy [10], the incidence of microalbuminuria and the risk of diabetic nephropathy in type 2 diabetes [11] and the likelihood of newly diagnosed diabetes mellitus [12]. The latest therapeutic guidelines have already incorporated all these evidences [13]–[18], and ACE inhibitors are kept on record as a promising component of polypills in primary prevention of large mortality diseases [19].

Motivated by the obvious effectiveness of ACE inhibitors, efforts have been made to associate ACE expression with cardiovascular pathologies to introduce personalized therapies. The clinical efficacy of ACE inhibitors appears to be genetically determined, as ACE inhibitors are less effective in African-American patients than in Caucasian patients [20]. Genetic studies have revealed that the expression of ACE is controlled by an insertion/deletion (I/D) polymorphism in the ACE gene, which results in an ACE expression that is approximately 50% higher in individuals with genotype DD than in those with genotype II [21]. Although some later reports downgraded the level of contribution of ACE genotype to ACE expression (about 20% [22], [23] or only 8% [24]), ACE genotype has been studied extensively as a major cardiovascular risk factor.

Recently, we have confirmed that the human serum albumin (HSA) is an endogenous ACE inhibitor [25]. HSA antagonized serum ACE activity with an IC_50_ value of 5.7±0.7 mg/mL [25], while physiological HSA concentrations were in the 35–52 mg/mL range in our studies. These data suggested that ACE is significantly inhibited by HSA, *in vivo*.

The goal of our study was to validate endogenous ACE inhibition in clinical studies. Recently we have found that endogenous ACE inhibitors [26] such as human serum albumin (HSA) [25] stabilize angiotensin converting enzyme (ACE) activity at a very low level [25], [26] independently of ACE expression levels. In accordance, ACE expression and ACE genotype had no effect on cardiovascular disease parameters in clinical studies reported here.

## Methods

### Ethical approval

All of the studies were approved by the Regional and Institutional Ethics Committee, Medical and Health Science Center, University of Debrecen, (UDMHSC REC/IEC number: 2894-2008) and by the Medical Research Council of Hungary. All of the patients involved gave their written informed consent.

### Patient's blood sample collection, serum and DNA isolation

Blood samples were collected from volunteers by using a standard aseptic technique. Native blood was incubated for 60 minutes at room temperature; serum fractions (separated by centrifugation at 1,500 *g* for 15 min) were stored at −20°C until further experiments. Genomic DNA was prepared from anticoagulated venous blood by using a DNA separation kit (Qiagen).

### ACE activity measurement

ACE activity was measured as originally described by Beneteau et al. [27] and modified by us [26]. In brief, ACE activity was determined with an artificial substrate (FAPGG, (*N*-[3-(2-furyl)acryloyl]-L-phenylalanylglycylglycine; Sigma-Aldrich) in a reaction mixture containing 25 mM HEPES (*N*-2-hydroxyethylpiperazine-*N*-2-ethanesulfonic acid), 0.5 mM FAPGG, 300 mM NaCl, and the desired dilution of serum, at pH 8.2. Measurements were performed in 96-well plates (Greiner-Bio One) at 37°C. Changes in optical density (340 nm) were measured at 5-min intervals for at least 90 min with a plate reader (NovoStar plate reader; BMG Labtech). Optical density values were plotted as a function of reaction time and fitted by linear regression. The fit and the data were accepted when *r^2^* was >0.90. ACE activity was calculated via the equation:
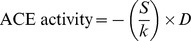
where *S* is the rate of observed decrease in optical density (1/min), *k* is the change in optical density upon the complete cleavage of 1 µmol of FAPGG, and *D* is the dilution of the serum. ACE activity is given in units where 1 U is equivalent to the cleavage of 1 µmol of FAPGG in 1 min. In some experiments, the reaction mixture also contained human serum albumin (HSA; Human BioPlazma Manufacturing and Trading).

### Measurement of human serum albumin concentration

Human serum albumin concentration was measured with bromocresol green (BCG, Dialab) diagnostic reagent according to the manufacturer's instructions. Briefly, 10 µl serum sample was added to 1 mL bromocresol green reagent (260 µM bromocresol green in 30 mM citrate buffer, pH 4.2), and the mixture was incubated for 10 minutes at room temperature. The absorbance of the mixture was measured at 546 nm against reagent blank in a spectrophotometer (U-2900, Hitachi), using disposable cuvettes. HSA concentration was calculated via the equation:
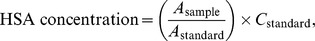
where A is the absorbance of mixture at 546 nm, C_standard_ is the HSA concentration of a diagnostic standard (Albumin standard, Dialab).

### Determination of ACE I/D polymorphism

**Figure 1:**
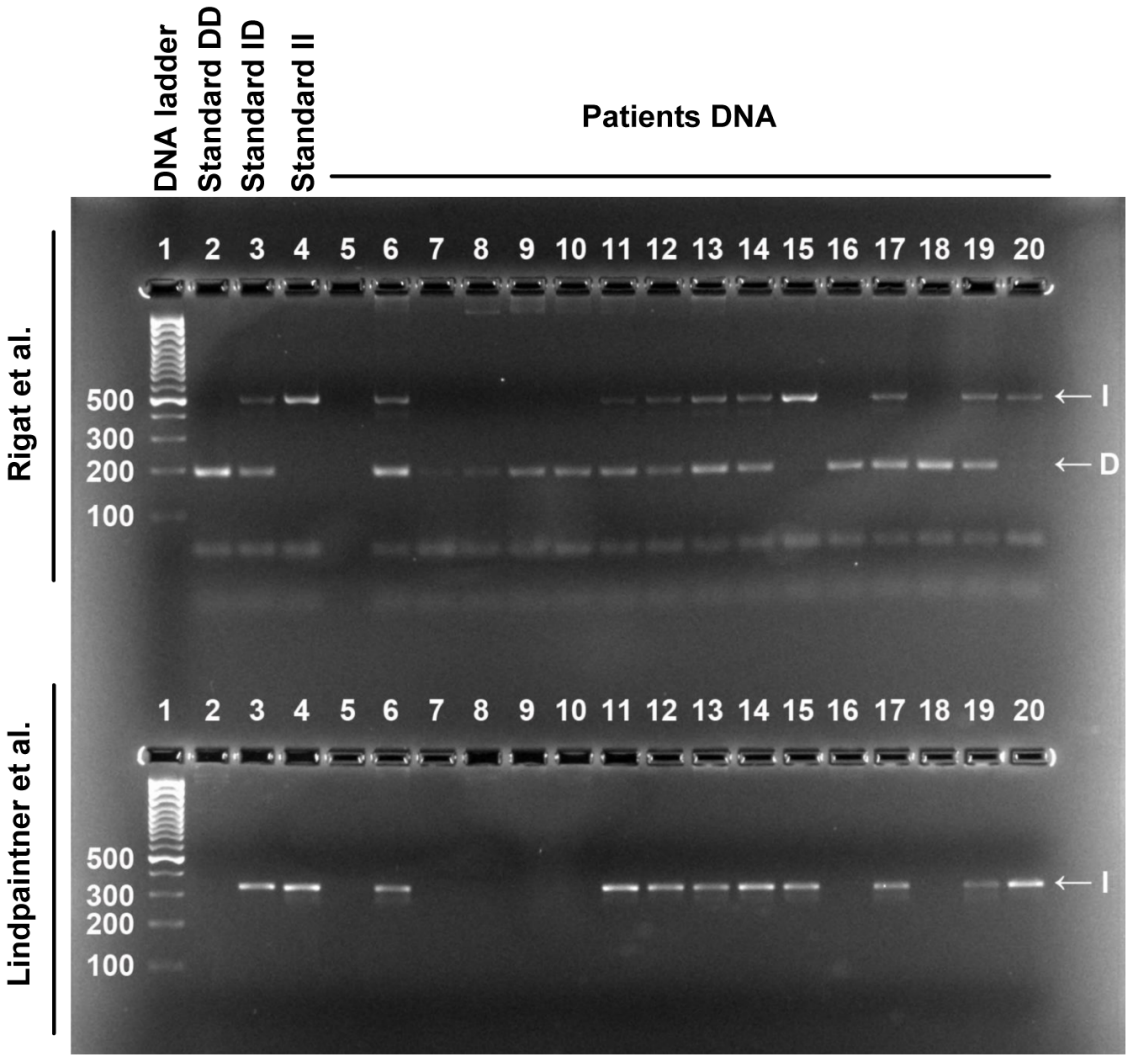
Determination of ACE genotype. ACE genotype was determined by PCR based assays. First, PCR was performed according to the method described by Rigat et al. (indicated). Amplified DNA with a size of 490 bp represented the I allele, while D allele resulted in a 190-bp band. The results were then verified by the method described by Lindpaintner et al. (indicated). Allele I resulted in a 335-bp amplicon in this case.

The insertion/deletion genotype (I/D) of ACE was determined by PCR amplification of alleles I and D on the basis of the standard protocol described by Rigat et al. [28]. The amplification products were separated on 5% polyacrylamide gels, and were visualized by ethidium-bromide staining. The presence of allele I or D resulted in a 490-bp or a 190-bp PCR product, respectively (Fig. 1). Insertion specific polymerase chain reaction was performed to avoid mistyping ID and DD genotypes using a protocol described by Lindpaintner et al. [29]. In this case the presence of allele I resulted in a 335-bp amplicon visualized on 3% agarose gel (Fig. 1).

### Measurement of serum ACE concentration

**Figure 2:**
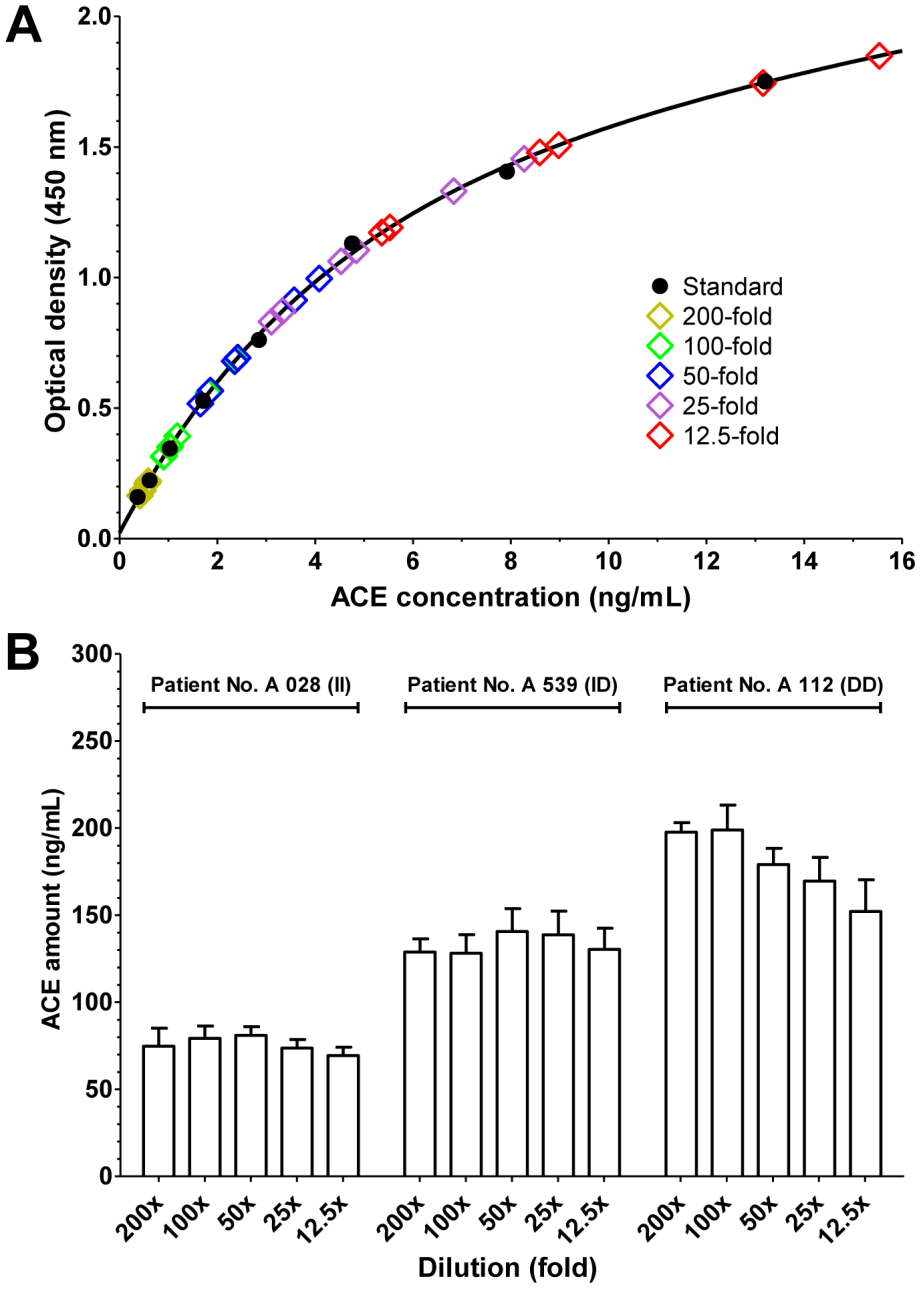
Determination of ACE concentration. Three patients were selected to represent all genotypes (II, ID and DD, indicated). Sera were diluted 12.5, 25, 50, 100 and 200-fold (indicated) to determine optimal conditions. ACE amount was determined in an ELISA assay. A set samples with known ACE concentrations (Standard, **A**) were also used. Values (optical density) were plotted as the function of the known ACE concentration and a calibration curve was defined (nonlinear fit, solid line, **A**). Concentrations of the serum samples were then calculated based on their position on the calibration curve. Each serum sample was determined two times. Average of the quadruple measurements is shown (three samples measured twice, resulting in 6, sometimes overlapping individual data point for each dilution, **A**). It was apparent from the individual measurements that dilutions at 200-fold have very low values, while the saturation of the binding sites resulted in a high scatter at dilutions below 50-fold (**A**). Although averaged values showed a tendency for under-estimating ACE concentrations at low dilutions, in the case of high expression levels (**B**), ACE concentration determination was remarkably accurate. Bars represent mean ± SD. No statistical differences were found by nonparametric ANOVA.

Serum ACE concentration was measured by a commercial human ACE ELISA development kit (R&D Systems) according to the manufacturer's instructions, with minor modifications. Briefly, enzyme-linked immunosorbent plates (Greiner Bio-One) were coated with 80 ng/well capture antibody, and the remaining binding sites were then blocked with reagent diluent (10 mg/mL bovine serum albumin (Sigma-Aldrich) in Dulbecco's phosphate-buffered saline solution (PBS, Gibco)). Diluted sera (in reagent diluent, 12.5–200-fold dilution) were added to the wells, and the antibody-antigen complexes were labeled with a biotinylated detection antibody (20 ng/well). 200-fold-diluted streptavidin-conjugated horseradish-peroxidase (kit component) was added to the wells. Finally, the amounts of complexes were detected with a substrate solution containing 0.3 mg/mL tetramethylbenzidine, 0.1 µM H_2_O_2_ and 50 mM acetic acid. The reaction was terminated after 20 min by the addition of 0.5 M HCl, and the optical density was measured at 450 nm. To determine the optimal conditions to measure ACE concentration in human sera with II, ID and DD genotypes a series dilutions were tested (12.5–200-fold, Fig. 2). The 100-fold dilution was found to be optimal: the values were in the linear phase (in contrast with dilutions below 50-fold dilutions) and were sufficiently high to measure. The ACE concentrations in the samples were measured at least three times to achieve a standard deviation of at most 15%.

### Calculation of ACE activity in the undiluted human sera

ACE activity in the undiluted sera was calculated according to the equation:
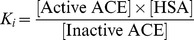
Where Ki is the inhibitory constant of HSA on ACE activity determined previously (83 µM) [25], [Active ACE], concentration of the active form of ACE; [Inactive ACE], concentration of the inhibited form of ACE; [HSA], concentration of human serum albumin, determined by colorimetric assay.

Taking into account that
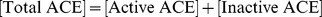
where [Total ACE] is determined by ELISA,

[Active ACE] can be calculated based on measured values as:
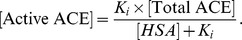
Activity of [Active ACE] was related to the measured ACE activity in the same patients at 20-fold dilution, where ACE activity was considered to be uninhibited by HSA [25], to estimate the ACE activity in the undiluted human sera.

### Statistical analysis

Statistical analysis was performed with Graphpad Prism 5.0 (GraphPad Software) by one-way ANOVA, one-way ANOVA with Dunnett's multiple comparison test and by the t-test. Differences were considered to be significant when *p*<0.05.

## Results

**Figure 3:**
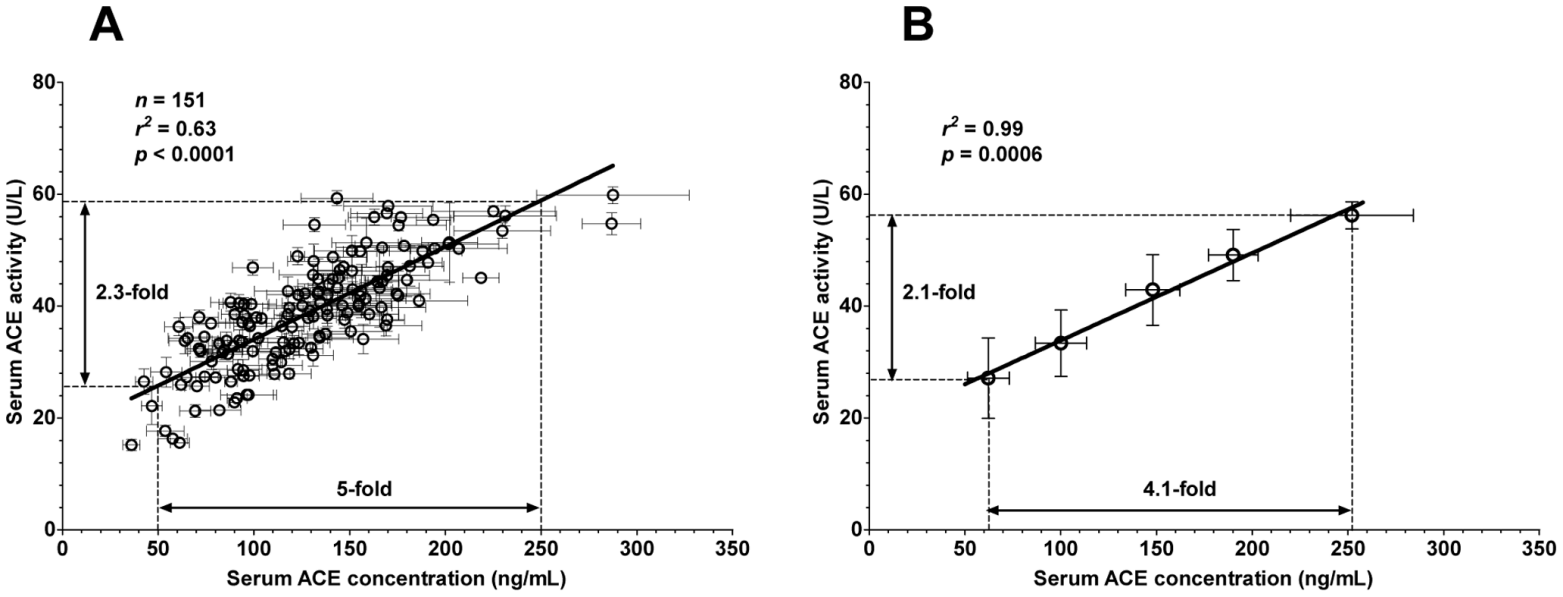
Incomplete correlation between serum ACE concentration and activity. Serum ACE concentration and specific ACE activity was determined in hypertensive patients (n = 151 patients). Serum ACE concentration was determined by an ELISA method using 100-fold diluted serum samples. Human recombinant ACE was used to construct calibration curves for each individual plate. Serum ACE expression was calculated based on these calibration curves. ACE activity was assessed at a 4-fold dilution by measuring the decomposition of the artificial substrate FAPGG. Serum ACE activity is shown as a function of the serum ACE concentration in the figures. Panel **A** shows the individual data, while patients were grouped according to their serum ACE concentration in panel **B**. Both graphs were fitted by a linear regression to reveal correlations between the values, and the parameters of the fits are shown. The magnitude of the differences in ACE concentration and ACE activity values are also shown. Symbols represent the mean (measurements were done at least 3-times), bars are SEM.

Plotting ACE activity as a function of the serum ACE concentration yielded a linear relationship in the individual patients (Fig. 3A), and in patient groups with different levels of endogenous ACE expression (Fig. 3B). However, a 5-fold increase in serum ACE concentration was only accompanied by a 2.3-fold increase in serum ACE activity based on the linear fit of the individual values (Fig. 3A). Similarly, the increase in serum ACE concentration from 62±11 ng/mL to 252±32 ng/mL (4.1-fold increase in expression, Fig. 3B) was accompanied by a limited (2.1-fold) increase in ACE activity (from 27±7 U/L to 56±2 U/L, Fig. 3B).

**Figure 4:**
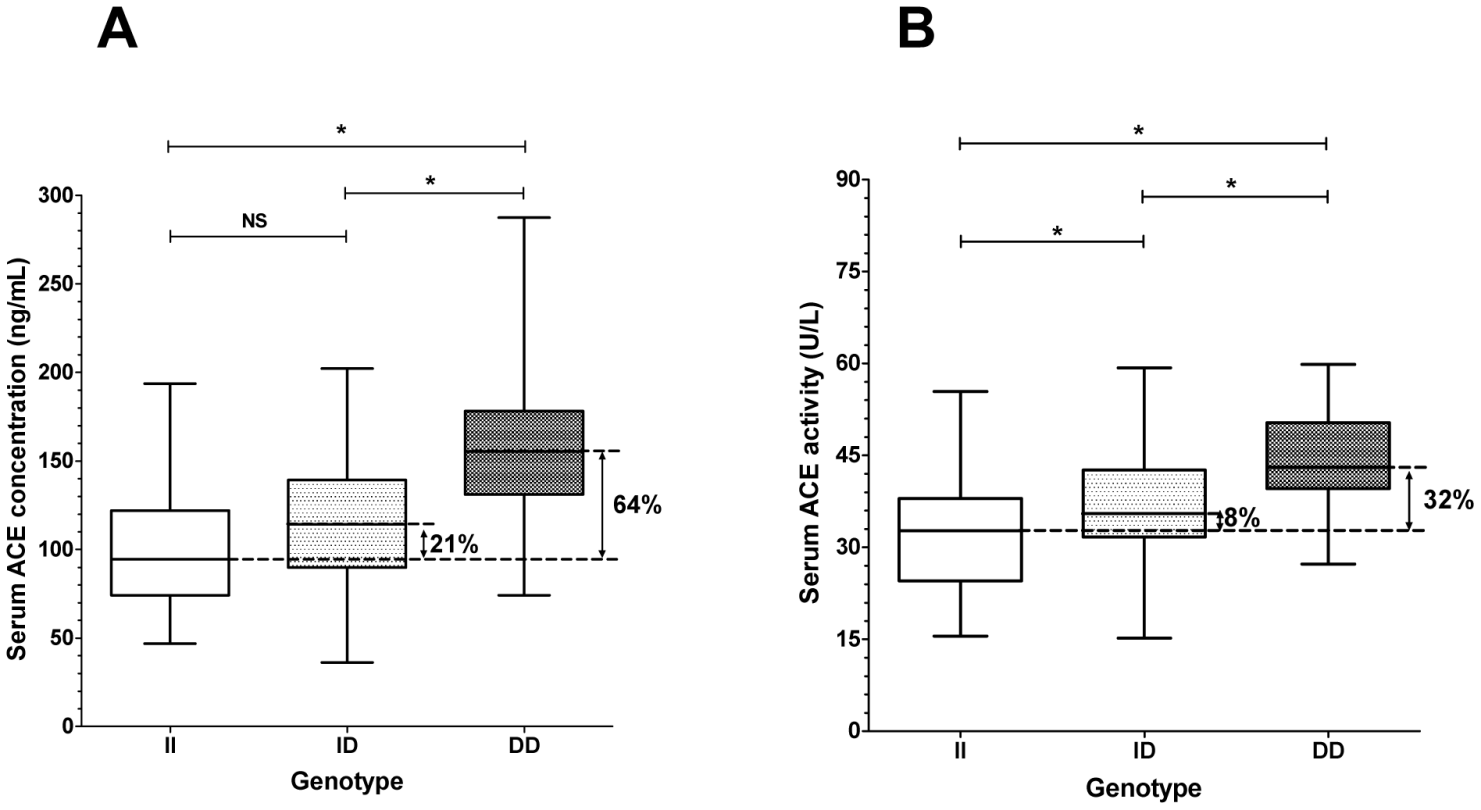
ACE genotype determines serum ACE concentration and activity. Serum ACE concentration, activity and genotype was determined as mentioned earlier (Figs. 3, 1). ACE concentration (**A**) and activity (**B**) in patients with different ACE genotypes (insertion, II, *n* = 28; deletion, DD, *n* = 52 and heterozygotes, ID, *n* = 71) are shown. The boxes indicate the interquartile range with median; whiskers are the minimum and the maximum values. Significant differences (1 way ANOVA, Bonferroni post-hoc test) are labelled by asterisks (NS, not significant).

Patients who were homozygous for the allele D (deletion) of the ACE gene displayed higher ACE concentrations in their sera than those who were homozygous for the allele I (Insertion, II = 47–194 ng/mL, median: 94.5 ng/mL, *n* = 28; ID = 36–202 ng/mL, median: 114.3 ng/mL, *n* = 71; DD = 74–288 ng/mL, median: 155.2 ng/mL, *n* = 52; Fig. 4A). The activity of ACE was also elevated in the DD homozygote patients (II = 15.6–55.4 U/L, median: 32.74 U/L, *n* = 28; ID = 15.2–59.3 U/L, median: 35.48 U/L, *n* = 71; DD = 27.3–59.8 U/L, median: 43.11, *n* = 52; Fig. 4B). Nonetheless, these data indicate that a 64% increase in serum ACE concentration results in only a 32% increase in ACE activity in patients with the DD genotype versus those with the II genotype.

**Figure 5:**
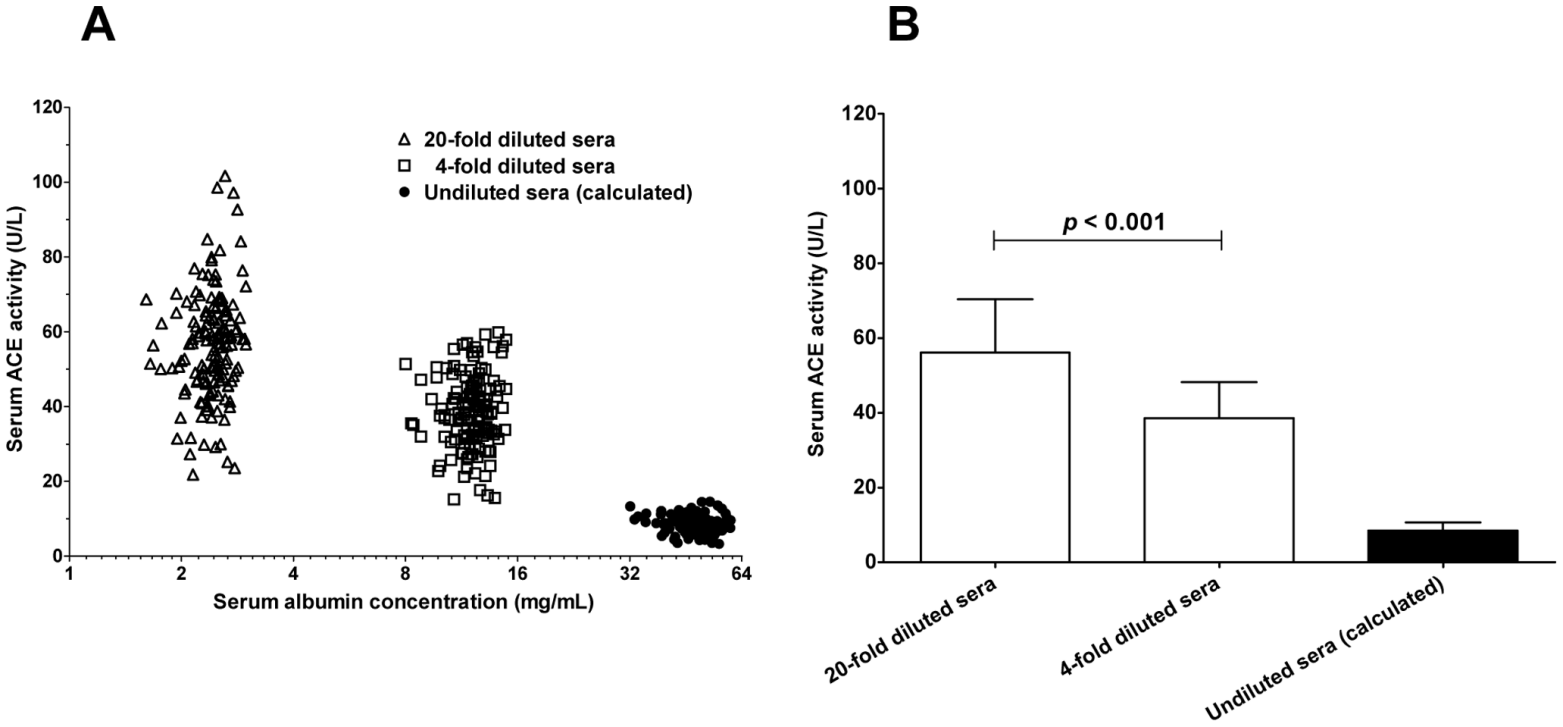
Serum ACE activity correlates with serum albumin concentration. Serum ACE activities (*n* = 151 patients) were determined at different dilutions (20-fold and 4-fold dilutions). The determined ACE activity values were plotted as a function of the actual serum albumin concentrations in the individual samples (**A**). Serum ACE activity values for undiluted sera were estimated based on the measured activity values at 20-fold dilution and the reported ACE-HSA inhibitory constant (IC_50_ = 5.7 mg/mL). These calculated ACE activity values were plotted as a function of the measured human serum albumin concentration in the individual serum samples (labelled as undiluted sera). Values are also given as the mean and SD of the individual determinations (**B**). There was a significant difference (unpaired t-test) between the values measured at 20-fold and 4-fold dilutions (indicated).

These data suggest that the ACE activity is modulated by additional factors besides the ACE concentration in the human serum [26]. Previous results suggested that serum albumin is an endogenous ACE inhibitor [25], [30], which has a concentration dependent effect on serum ACE activity. An effort was made to investigate the relationship between serum ACE activity and serum albumin concentration in human sera under *in vitro* conditions. Apparent specific ACE activity was higher when serum albumin concentration was low due to the dilution of the sera (Fig. 5). In particular, apparent specific ACE activity decreased from 56.13±1.17 to 38.55±0.78 U/L when HSA concentration increased from 2.42±0.02 mg/mL to 12.12±0.12 mg/mL. The stability constant (IC_50_ value) of the human serum ACE-HSA interaction was 5.7±0.7 mg/mL in a previous study in our laboratory [25], suggesting that the ACE activity is 8.47±0.18 U/L (calculated values) in the presence of the measured 48.46±0.46 mg/mL HSA concentrations in human serum samples used for the analysis (Fig. 5).

**Figure 6:**
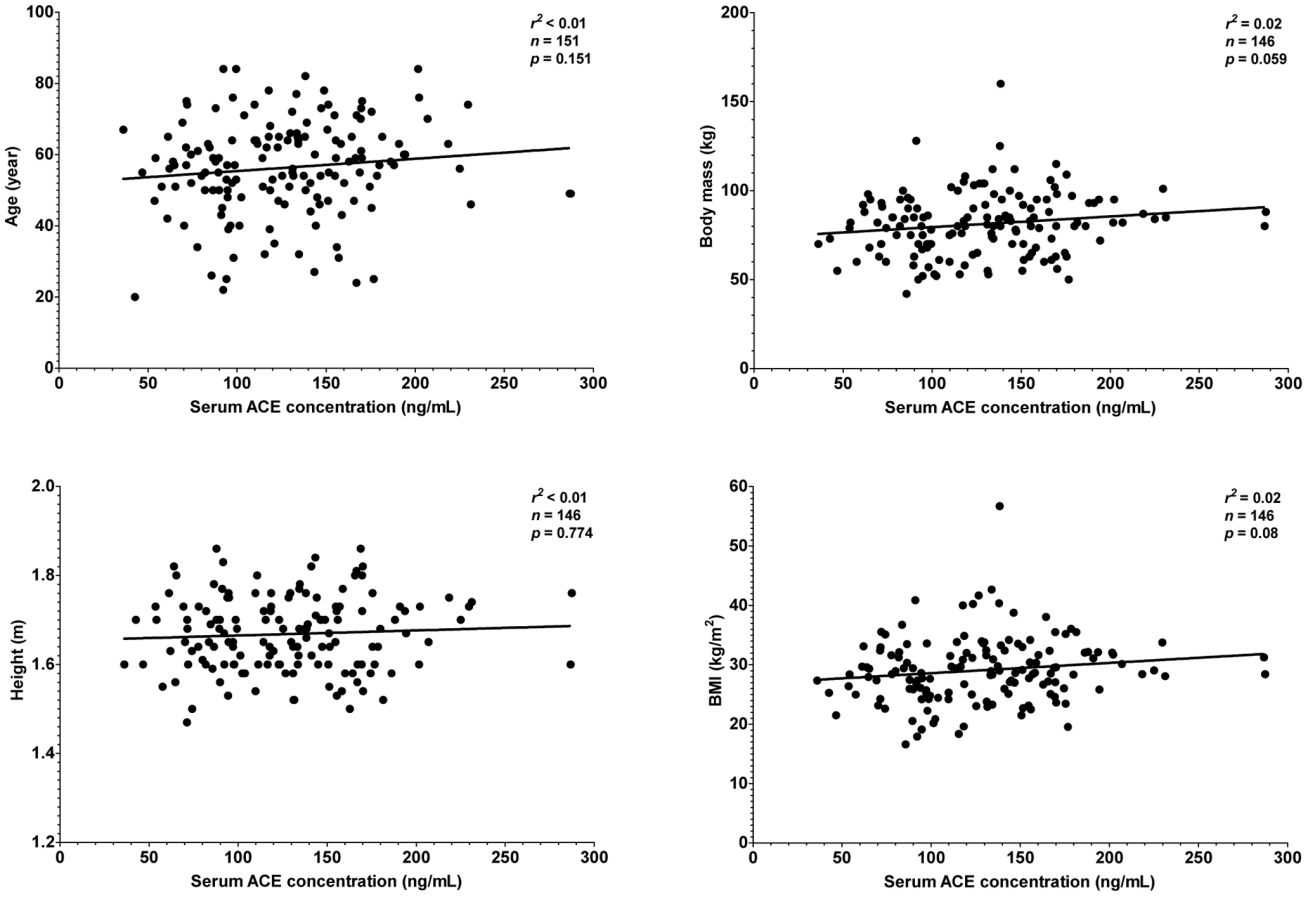
There is no relationship between morphometric parameters and serum ACE concentration. Age, body mass, height and body mass index of the patients involved in the studies (*n* = 151) are plotted as the function of the measured serum ACE concentration. Points were fitted by a linear regression to reveal the potential correlation between the plotted parameters. Parameters of the fitting are shown in each graph. The normal range for BMI is 18.5–25 kg/m^2^.

**Figure 7:**
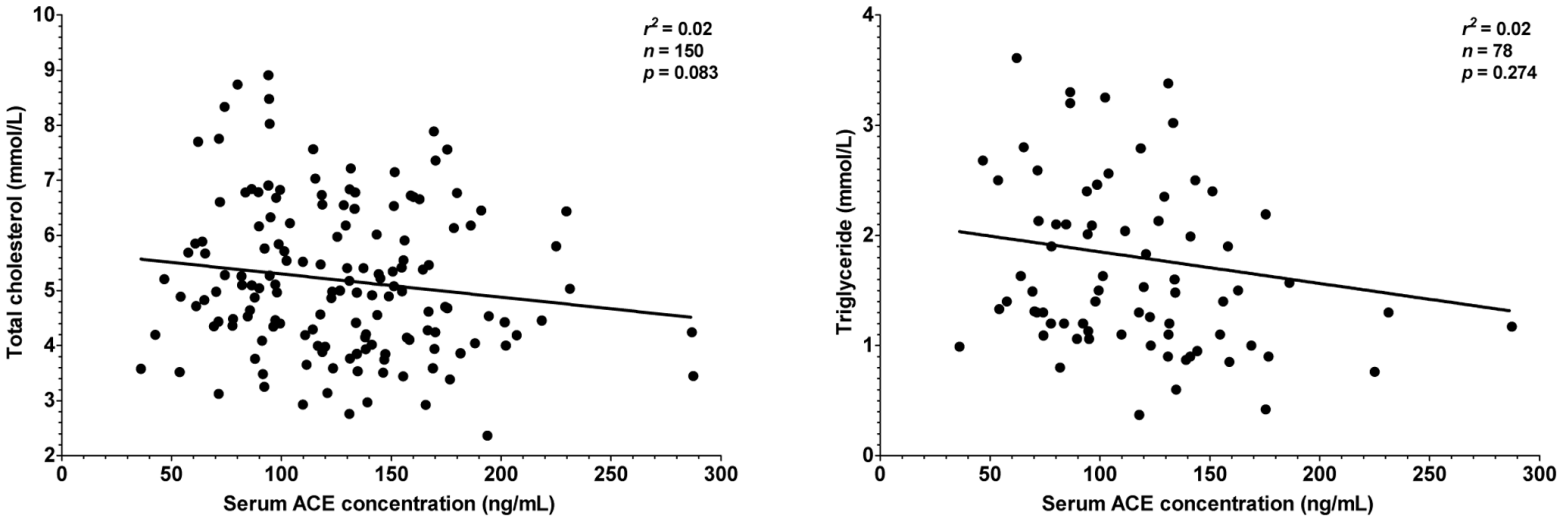
There is no relationship between serum lipid levels and serum ACE concentration. Total cholesterol and triglyceride levels are plotted as a function of the serum ACE concentration (*n* = 151 patients). Points were fitted by a linear regression to reveal the potential correlation between the plotted parameters. Parameters of the fitting are shown in each graph. Normal rage for total cholesterol is lower than 5.2 mmol/L for triglyceride is lower than 1.7 mmol/L.

**Figure 8:**
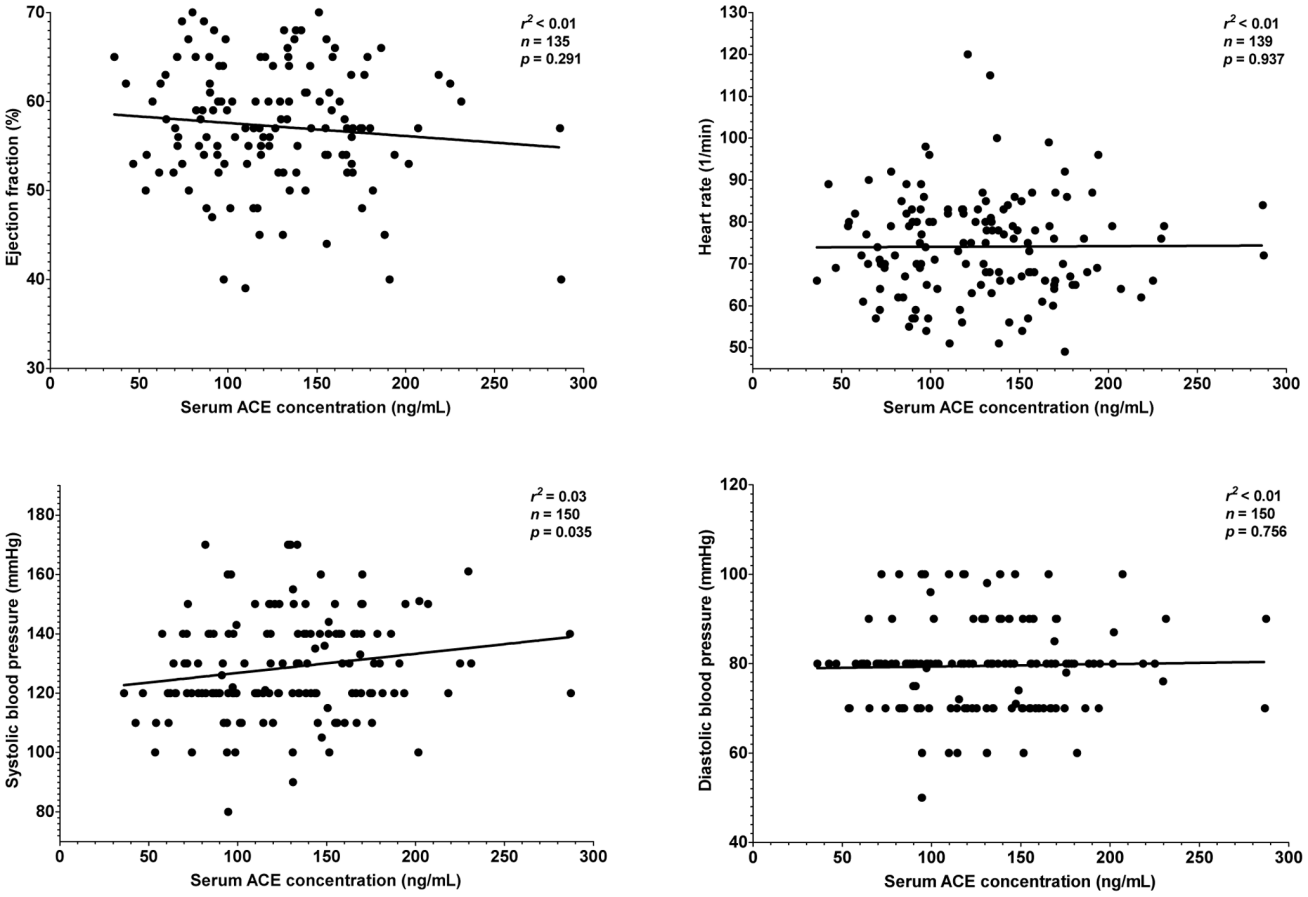
There is no relationship between cardiovascular parameters and serum ACE concentration. Cardiovascular parameters (ejection fraction, heart rate, systolic and diastolic blood pressure) were plotted as a function of the serum ACE concentration (*n* = 151 patients). Points were fitted by a linear regression to reveal the potential correlation between the plotted parameters. Parameters of the fitting are shown in each graph. Ejection fraction is normally above 50%, heart rate is in the range of 60–100/min, systolic and diastolic blood pressure is in the range of 90–140 and 60–90 mmHg.

**Figure 9:**
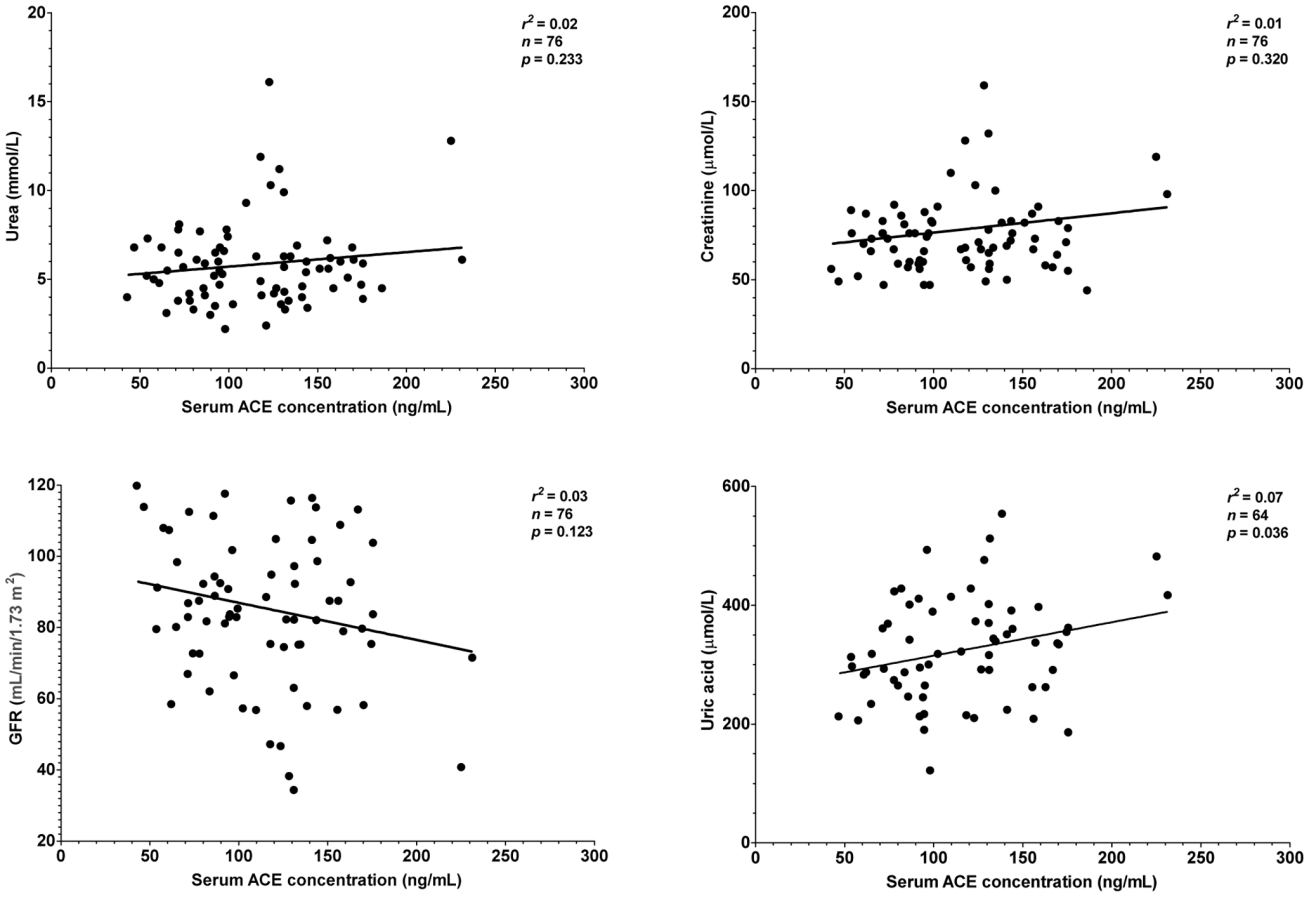
There is no relationship between parameters of renal function and serum ACE concentration. Renal function was assessed by the means of urea, creatinine and uric acid concentrations in the sera and by the glomerular filtration rate (GFR) values. Values were plotted as a function of the serum ACE concentration (*n* = 151 patients). Points were fitted by a linear regression to reveal the potential correlation between the plotted parameters. Parameters of the fitting are shown in each graph. Normal ranges for the values are: urea, 3.6–7.2 mmol/L, creatinine, 44–97 µmol/L (female) and 62–106 µmol/L (male), GFR, above 90 mL/min/1.73 m^2^, uric acid, 140–340 µmol/L (female) and 220–420 µmol/L (male).

**Figure 10:**
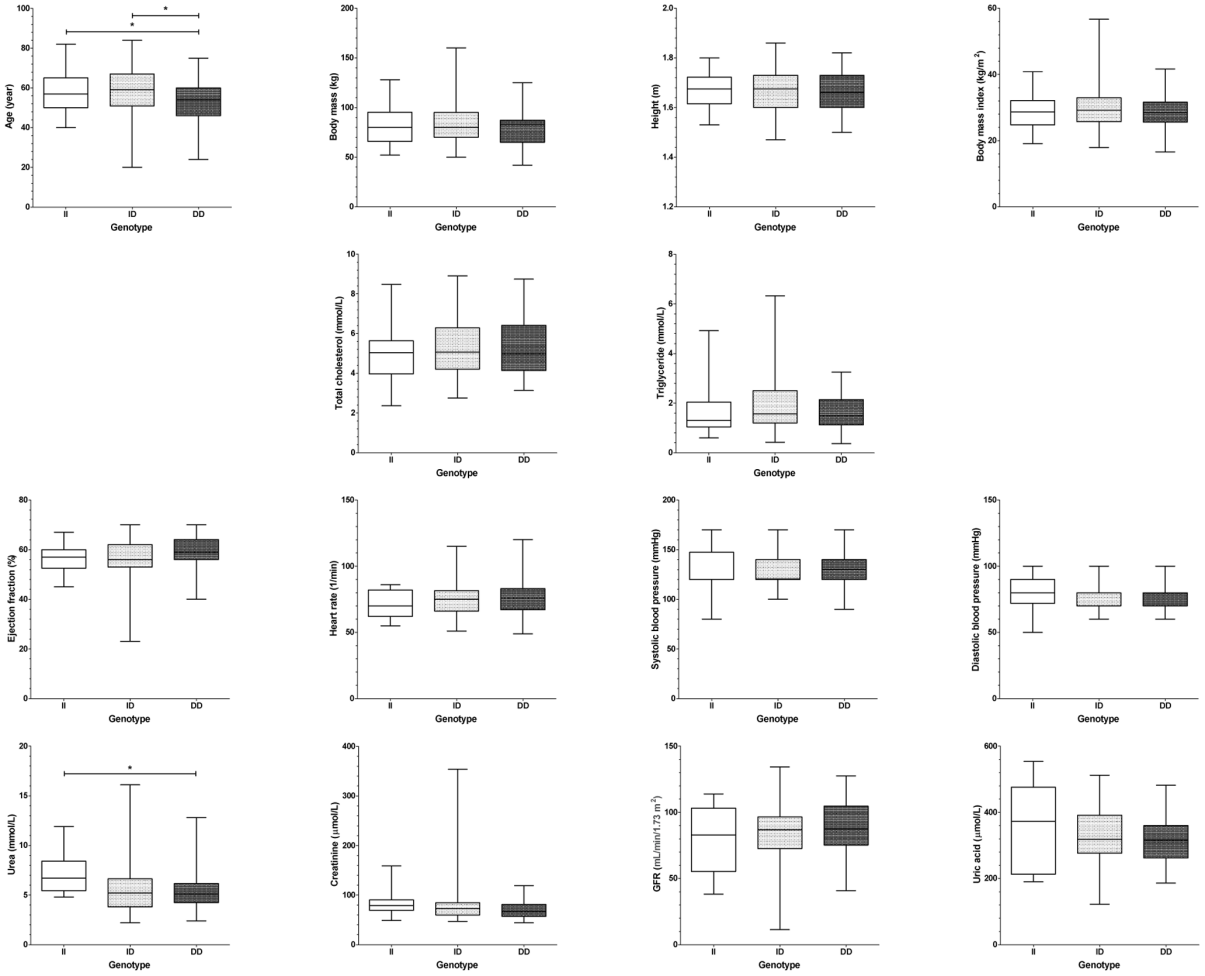
Correlation of patient's parameters with ACE I/D genotype. Various parameters (age, body mass, height, body mass index, total cholesterol, triglyceride, ejection fraction, heart rate, systolic and diastolic blood pressure, urea, creatinine, glomerular filtration rate and uric acid) were correlated to the genotype of the patients (insertion, II, *n* = 28; deletion, DD, *n* = 52 and heterozygotes, ID, *n* = 71). The boxes indicate the interquartile range with median; whiskers are the minimum and the maximum values. Significant differences (1 way ANOVA, Bonferroni post-hoc test) are labelled by asterisks.

It was tested whether this endogenous suppression of serum ACE by HSA is sufficient to compensate for different expression levels in hypertensive patients where therapeutic ACE inhibition is particularly effective. Patients without prescribed ACE inhibitory drugs were recruited (n = 151 patients, Table 1). Serum ACE concentration did not correlated with morphometric parameters such as age, body mass, height or body mass index (Fig. 6). Serum ACE concentration did not show correlation with lipid parameters, such as total cholesterol and triglyceride concentration (Fig. 7). Cardiovascular parameters (ejection fraction, heart rate, systolic and diastolic blood pressure) were again independent of serum ACE concentration (Fig. 8), based on the inaccurate fit (represented by the low *r^2^* values). Parameters of the renal function (serum urea, creatinine, uric acid and glomerular filtration rate) again showed a significant scatter (see low *r^2^* values) and suggested no correlation with serum ACE concentration (Fig. 9). Correlation of these values was also tested with the genotype of the patients, which significantly affected ACE expression (Fig. 4A). No correlation was found between clinical parameters and ACE genotype (Fig. 10), similarly to the individual ACE expression levels (Fig. 6–9).

**Table 1:** Clinical parameters and the genotype of the patients involved in the study.

		II	ID	DD	All patients
Number of patients		28	71	52	151
Gender (female/male)		12/16	33/38	36/16	81/70
Age		58±11	58±14	52±13	56±13
Body mass Index (kg/m2)		28.9±5.6	29.2±5.9	29.0±5.0	29.0±6.0
Smoker		4 (14%)	15 (21%)	7 (13%)	32 (21%)
Diabetes mellitus	IDDM	2 (7%)	3 (4%)	3 (6%)	8 (5%)
	NIDDM	6 (21%)	13 (19%)	8 (15%)	27 (18%)
Dyslipidemia		19 (68%)	48 (68%)	32 (62%)	99 (66%)
Total cholesterol (mmol/L)		4.96±1.34	5.21±1.38	5.28±1.39	5.19±1.36
Ejection fraction (%)		57±6	56±8	59±7	57±7
Heart rate (1/min)		71±10	75±12	75±13	74±12
Systolic blood pressure (mmHg)		130±21	128±16	128±16	129±17
Dyastolic blood pressure (mmHg)		81±12	80±9	78±10	80±10
Urea (mmol/L)		7.3±2.3	5.6±2.7	5.4±1.9	5.8±2.3
Creatinine (µmol/L)		85±29	82±52	71±17	77±38
Glomerular filtration rate (mL/min/1.73 m2)		75±21	87±25	85±12	84±20
Uric acid (µmol/L)		356±126	313±101	313±72	319±94
ACE amount (median (min, max), U/L)		95 (47, 194)	114 (36, 202)	155 (74, 288)	127 (36, 288)
ACE activity (median (min, max), ng/mL)		33 (16, 55)	35 (15, 59)	43 (27, 60)	38 (15, 60)
Used antihypertensive drugs:					
Diuretics		15 (54%)	23 (32%)	16 (31%)	54 (36%)
Clopamide		0	1 (1%)	0	1 (0.5%)
Hydrochlorothiazide		10 (36%)	13 (19%)	13 (25%)	36 (24%)
Indapamide		0	3 (4%)	0	3 (2%)
Spironolactone		3 (11%)	3 (4%)	3 (6%)	9 (6%)
Amiloride		2 (7%)	3 (4%)	0	5 (3%)
β-Blockers		21 (75%)	51 (72%)	32 (62%)	104 (68%)
Betaxolol		0	1 (1%)	0	1 (0.5%)
Bisoprolol		11 (40%)	24 (34%)	19 (37%)	54 (36%)
Carvedilol		2 (7%)	5 (7%)	2 (4%)	9 (6%)
Metoprolol		2 (7%)	5 (7%)	2 (4%)	9 (6%)
Nebivolol		6 (21%)	16 (23%)	9 (17%)	31 (21%)
α1-Antagonists		3 (11%)	4 (6%)	2 (4%)	9 (6%)
Doxasosin		2 (7%)	3 (4%)	0	5 (3%)
Prasosin		1 (4%)	1 (1%)	2 (4%)	4 (3%)
ACE-Inhibitor		0	0	0	0
Angiotensin receptor blockers		17 (61%)	31 (44%)	22 (42%)	70 (46%)
Irbesartan		6 (21%)	13 (19%)	11 (21%)	30 (20%)
Losartan		7 (25%)	15 (21%)	5 (10%)	27 (18%)
Telmisartan		1 (4%)	2 (3%)	4 (8%)	7 (5%)
Valsartan		3 (11%)	1 (1%)	2 (4%)	6 (4%)
Renin-Inhibitor		0	0	0	0
Ca-channel blockers		11 (39%)	20 (28%)	13 (25%)	44 (29%)
Amlodipine		11 (39%)	19 (27%)	11 (21%)	41 (27%)
Dilthiazem		0	1 (1%)	1 (2%)	2 (1%)
Verapamil		0	0	1 (2%)	1 (0.5%)
Imidazoline-I agonisms		2 (7%)	1 (1%)	2 (4%)	5 (3%)
Rilmenidin		2 (7%)	1 (1%)	2 (4%)	5 (3%)
Statin therapy		13 (46%)	36 (51%)	23 (44%)	72 (48%)

## Discussion

Angiotensin converting enzyme (ACE) is one of the most frequently targeted enzymes in the everyday medical practice. ACE inhibitors are the fifth most described drugs and represent a cornerstone in the treatment in cardiovascular diseases (hypertension, heart failure) [13]–[18]. Surprisingly, we have confirmed recently [25], that serum albumin is an endogenous ACE inhibitor [31], which can inhibit ACE in a concentration dependent fashion (IC_50_ value is 5.7±0.7 mg/mL on human serum ACE in our studies [25]) similarly to the prescribed ACE inhibitory drugs.

We hypothesized here that endogenous ACE inhibitors [26], [32]–[45], such as human serum albumin [25] can provide an endogenous protection against dysregulation of ACE. Our recent data suggest that serum ACE activity is suppressed by endogenous ACE inhibitors [26], such as serum albumin [25], providing a protection against higher ACE expression levels in human serum [25].

Determination of ACE concentration was done by a kit manufactured by R&D Systems here. Since we did not find relevant publications using this kit to determine serum ACE, we have performed a detailed analysis of its reproducibility and made an effort to optimize the conditions. It appeared that the determinations are remarkably reproducible in a wide range of serum dilutions (12.5–200-fold). Nonetheless, 100-fold dilution appeared to be optimal (measurable values using limited volumes of serum samples). Values determined by this kit appeared to be somewhat lower than that published before (400–584 ng/mL) [23], [46], [47]. The reason of the differences can be related to the purity and quality of the standards used to quantitate concentrations. Irrespectively to the differences in the values determined by different methods, it appeared that our method results in an accurate determination of the differences in ACE concentration. These concentrations correlated by the genotype of the individuals (about 64% higher ACE concentration in patients with DD genotype compared to patients with II genotype).

ACE insertion-deletion (I/D) polymorphism was identified more than 20 years ago [21], and it was postulated that this polymorphism is responsible for 20–50% of the interpersonal variability in circulating ACE expression levels. In accordance, we found a wide range in serum ACE expression in human sera (from 47 to 288 ng/mL) and patients with DD genotype had 64% higher ACE expression than patients with II genotype. It was proposed that the differences in ACE expression related to ACE I/D polymorphism may play a role in the occurrence of myocardial infarction, coronary artery disease, coronary artery calcification, heart failure and hypertension. No such correlation was found here, in accordance with a meta-analysis of more than 30,000 individuals which concluded that ACE genotype is without effects on blood pressure, and is not associated with an increased risk of myocardial infarction, ischemic heart disease or ischemic cerebrovascular disease [48].

In the present study, we directly tested the relationship between ACE expression and activity in sera obtained from hypertensive patients. It was found that hypertensive patients with DD genotype had 64% higher serum ACE concentrations than in those with II genotype, while the difference in ACE activities was only 32%. This suggested that the effects of higher ACE expression are buffered. These findings are in accordance with the observation that local angiotensin I to angiotensin II conversion and the angiotensin II/I ratio are independent of the I/D genotype in the human forearm [49].

This apparent lack of correlation between cardiovascular parameters and ACE expression (genotype) is particularly interesting in light of the clinical effectiveness of ACE inhibitors for the same cardiovascular diseases, as evidenced by several large-scale trials, and as accepted by the published guidelines [4]–[18]. Several recent reviews evaluating the potential role of an increased ACE expression in cardiovascular disease concluded that not the expression (and genotype), but the actual ACE activity is important [50].

Important to note, that it is rather complicated to accurately determine serum ACE activity. In particular, earlier reports suggested that dilution of the serum has a significant effect on the apparent ACE activity values [32]. To eliminate this effect a high dilution factor was suggested during the determinations [51]. In a recent work we have identified the serum albumin as an endogenous ACE inhibitor, with an IC_50_ value (5.7±0.7 mg/mL) [25]. It was found that the apparent serum ACE activity is higher and the effect of dilution is missing when serum albumin is removed from the human sera [25], [26].

The major novelty of the present work is to show that endogenous inhibitors [26], such as HSA [25] suppress ACE activity in a clinical setup. Serum ACE activity did not correlate perfectly with the ACE concentration. A 4.1-fold increase in ACE concentration resulted in a 2.1-fold increase in ACE activity in the patient's sera, suggesting that serum ACE activity is regulated. Additional factors than the concentration of the enzyme contribute to the ACE activity. Moreover, this factor suppressed the effects of the different ACE expression levels on ACE activity, suggesting the existence of an endogenous ACE activity stabilizing mechanism.

This effect may be clinically significant when the circulating ACE level is elevated in consequence of genetic factors (such as the ACE I/D polymorphism) or diseases. A point mutation in the stalk region of the ACE gene resulted in a 5-fold elevated circulating ACE concentration [52]. This mutation affected at least eight families, but there were no ACE-related clinical abnormalities or hypertension. An other mutation that was accompanied by a 13-fold elevation of the serum ACE concentration was also without increased occurrence of cardiovascular disease [53]. Some other mutations of the ACE gene are known to be coupled with a mildly elevated ACE concentration, but again without an enhanced incidence of cardiovascular diseases [54]–[57]. All of these observations suggest that substantial differences in ACE concentrations are well tolerated *in vivo*. This tolerance can be explained by the observation that ACE activity is stabilized at a low level by endogenous factors [25], [26].

Unfortunately it was not possible to directly determine ACE activity in the undiluted human sera. ACE activities were measured at a 4-fold dilution because serum absorbed light significantly at 340 nm, where exogenous substrate conversion is detected. Nonetheless, ACE activity was estimated in undiluted human serum by measuring the serum albumin and ACE concentrations, ACE activity at 20-fold dilution and using the previously determined inhibitory constant for HSA on human serum ACE (IC_50_ = 5.7±0.7 mg/mL) [25]. According to these measured values and the calculations ACE activity is suppressed to 8.5±2.2 U/L by the physiological concentration of HSA in the undiluted human serum samples. Moreover, this ACE activity is only the maximal value (representing the isolated effect of HSA). Other proposed endogenous ACE inhibitory molecules may further suppress ACE activity, *in vivo* [26].

The measured concentrations of ACE (about 127 ng/mL) and serum albumin (about 48 mg/mL) in human serum samples suggest that differences in ACE concentration may be buffered by endogenous inhibitors [26], in particular by HSA [25]. Serum albumin is present at concentration of 10-fold higher than the IC_50_ value for HSA to inhibit ACE suggesting that the majority of ACE is in its HSA bound (inhibited) form. Free HSA concentration is negligibly affected by ACE binding, several orders of magnitude differences in ACE activity can be theoretically “buffered” by binding to HSA.

It needs to be mentioned that HSA is only one of the proposed endogenous ACE inhibitors. Existence of endogenous ACE (called kininase II at this time) inhibitors were proposed as early as 1979. Besides to Klauser et al., who reported first that serum albumin inhibits the ACE [30] Ryan et al. reported the existence of small (<10 kDa) molecular weight components of human sera [39]. Later studies identified substrate analogues (angiotensin I [45] and substance P [44]) able to inhibit ACE. Lieberman et al. reported an ACE inhibitor with an apparent molecular mass of >50 kDa [32], Ikemoto et al. reported an inhibitor with an apparent molecular mass of >10 kDa [38]. Both low and high molecular weight ACE inhibitors were shown in the rat lung [33] or in the human sera [58]. Peptides of the tryptic lysate of human plasma [35], [36], human serum albumin [59], bovine alphaS2-casein [43] and some types of honey were also found to inhibit ACE [41].

Taken together, it appears that HSA (and/or its fragments) has an effect on ACE activity, *in vivo*. On the other hand HSA is probably not the sole endogenous inhibitor of ACE, according to these reports, making it hard to estimate its contribution. We proposed a hypothesis, which was based on the effect and concentration response of HSA [25], which in itself appeared to be sufficient to explain the findings. This hypothesis was indirectly tested in this manuscript and other endogenous ACE inhibitors may also have contributed to the features described here.

The observed suppression of serum ACE activity provides basis for intriguing consequences. One of them is that physiological angiotensin II concentrations may be determined by angiotensin II elimination. In this case activation of RAAS, which is a cornerstone of cardiovascular diseases (such as hypertension and heart failure) may occur by the decreasing efficiency of angiotensin II elimination as has been shown in an accompanying clinical paper [60]. As a matter of fact, one of the best-characterized enzymes involved in angiotensin II elimination is ACE2 [61], activation of which is known to protect against hypertension, myocardial fibrosis, remodeling [62] and renal impairment [63].

Another implication is that the levels of these endogenous inhibitors (including HSA) may affect the function of the RAAS through modulating ACE activity. Our data suggest that ACE is well suppressed as long as the HSA concentration is at least ∼30 mg/mL. Indeed, postoperative infusion of HSA frequently evokes hypotension in patients receiving ACE inhibitor therapy [64].

In summary, our data suggest that human serum ACE activity is suppressed at a low level (buffered) by endogenous inhibitors [26], such as serum albumin [25] and probably other low molecular weight inhibitors. This suppression of serum ACE activity suggests that angiotensin II synthesis may be limited, in which case angiotensin II elimination may have a significant physiological role [60]. Moreover, clinical data provided here suggest that this suppression of physiological ACE activity may contribute to the tolerance of different ACE expression levels, such as determined by ACE I/D polymorphism.
